# Autonomous and In Situ Ocean Environmental Monitoring on Optofluidic Platform

**DOI:** 10.3390/mi11010069

**Published:** 2020-01-08

**Authors:** Fang Wang, Jiaomeng Zhu, Longfei Chen, Yunfeng Zuo, Xuejia Hu, Yi Yang

**Affiliations:** 1 Key Laboratory of Artificial Micro/Nano Structure of Ministry of Education, School of Physics and Technology, Wuhan University, Wuhan 430072, China; wangfang2200@whu.edu.cn (F.W.); zhujiaomeng@whu.edu.cn (J.Z.); coiyhm@whu.edu.cn (L.C.); zuoyf@whu.edu.cn (Y.Z.); huxuejiawuda@whu.edu.cn (X.H.); 2 Shenzhen Research Institute, Wuhan University, Shenzhen 518000, China

**Keywords:** optofluidics, ocean monitoring, colorimetric method

## Abstract

Determining the distributions and variations of chemical elements in oceans has significant meanings for understanding the biogeochemical cycles, evaluating seawater pollution, and forecasting the occurrence of marine disasters. The primary chemical parameters of ocean monitoring include nutrients, pH, dissolved oxygen (DO), and heavy metals. At present, ocean monitoring mainly relies on laboratory analysis, which is hindered in applications due to its large size, high power consumption, and low representative and time-sensitive detection results. By integrating photonics and microfluidics into one chip, optofluidics brings new opportunities to develop portable microsystems for ocean monitoring. Optofluidic platforms have advantages in respect of size, cost, timeliness, and parallel processing of samples compared with traditional instruments. This review describes the applications of optofluidic platforms on autonomous and in situ ocean environmental monitoring, with an emphasis on their principles, sensing properties, advantages, and disadvantages. Predictably, autonomous and in situ systems based on optofluidic platforms will have important applications in ocean environmental monitoring.

## 1. Introduction

The ocean is a vast repository of resources for human society and an essential material foundation for the sustainable development of society and environment. However, with continuous development and utilization of oceans by human beings, the ocean ecological environment has been gradually destroyed, and the marine ecological system has been severely damaged. Marine natural disasters, such as red tides and tsunamis, occurred frequently, bringing substantial economic losses and social impacts. Therefore, it is urgent to protect the ocean environment. Ocean environmental monitoring is an important link in protecting the marine environment. Developing the technology of ocean environmental monitoring is of great significance for the early predicting of marine disasters, preventing and reducing harmful disasters [1,2].

Recently, increasing attention has been paid to the autonomous and in situ ocean monitoring. The primary parameters of ocean environmental monitoring include nutrients (e.g., nitrate, phosphate, silicate), pH, DO, and heavy metals [3,4,5,6,7,8,9]. Traditional ocean environmental monitoring mainly relies on manual processing of a large amount of seawater samples and laboratory analysis, which consists of multiple steps (e.g., sampling, transport, pretreatment, instrument analysis) and is rather costly and time-consuming. Besides, the obtained results may be inaccurate since the seawater samples could undergo unexpected reactions during the long-time operation. The relatively low representative and timeliness make it challenging to meet the requirements of rapid detection of marine indicators. Moreover, bulky and expensive high tech instruments and professional personnel are required for traditional marine monitoring. As a result, it is difficult to support ocean environmental monitoring for early warning and management.

Optofluidics is a relatively new technology field that synergistically integrates the optics and microfluidics; it provides many particular characteristics for enhancing the sensing performance and the minimization of systems [10,11,12,13,14,15,16]. Fluid is the natural carrier of various micro-nano particles (including chemical macromolecules and biomolecules). Together with the fluid, particles can directly flow into the optofluidic system, where chemical reactions can complete quickly. Optofluidics has the potential to easily detect various optical parameters with high sensitivity and accuracy, such as refractive index, fluorescence, and absorbance. It avoids the complicated processes in traditional detection methods. Besides, the most favored materials for optofluidic chips are polydimethylsiloxane (PDMS) [15] and polymethyl methacrylate (PMMA), both of them are cheap and easily replaceable. These advantages of high integration, sensitivity and accuracy, and low cost make the optofluidic technology widely used in environmental monitoring [5,17] and biochemical sensing.

The development of an autonomous and in situ ocean environmental monitoring system is of high priority in oceanographic research [18]. Optofluidics bring new ideas for the miniaturization of detection systems and has been increasingly considered as a powerful technology to realize ocean environmental monitoring. At present, although a few integrated systems based on optofluidic platform have been reported, a detailed review on its applications on ocean environmental monitoring is absent. Here, we describe the optofluidics-based autonomous monitoring of nutrients, pH, DO, and heavy metals in oceans, with a focus on their principles, sensing properties, advantages, and disadvantages compared with other reported ocean monitoring methods. Much attention has been paid to the most commonly used colorimetric/spectrophotometric detection method, as it has the capability of integrating all of the processes involved in the analysis into a small chip. At last, we forecast that an autonomous and in situ system based on optofluidic platforms will play important roles in the development of ocean environmental monitoring.

## 2. Nutrients

Soluble inorganic nitrogen, phosphorus, and silicate in seawater are essential nutrients for the survival of marine organisms. A moderate amount of nutrients in seawater promotes the growth of biology and microorganisms, while inadequate nutrients restrict the growth of phytoplankton and excessive nutrients are prone to cause eutrophication and even further lead to harmful algal blooms, extreme depletion of DO, and even death of aquatic organism [19,20,21]. Generally, the nitrite concentration in seawater is very low and stable. A too high concentration of nitrite or dramatic changes of nitrite concentration often indicate changes in the ocean environment. Phosphate is an important indicator of eutrophication, and the distribution of silicate affects the community structure of planktonic algae. Accurate quantification of such nutrients in oceans is essential for comprehending the dynamics of marine ecosystems and forecasting the occurrence of harmful red tides [5].

Various techniques, such as colorimetry [22,23,24,25,26,27,28,29,30,31,32,33,34], chemiluminescence [35,36], fluorimetry [37,38,39,40], electrochemistry [41,42,43], and chromatography [44,45,46], have been proposed for nutrient determination. Among them, the colorimetric method using chromogenic agents is one of the most favored detection approaches due to its stability, excellent detection limits, simplicity, high cost efficiency, and analytical feasibility. It operates by adding a color-developing agent to samples, and the absorbance spectrum of the product solution is proportional to the concentration of the nutrient based on the Beer–Lambert law:

(1) $$A\left(\lambda \right)=-\mathrm{lg}\left(\frac{I}{I_{0}}\right)=\varepsilon \left(\lambda \right)\mathrm{cl}$$

where, A(λ) is the absorbance of the solution at a wavelength λ, I_0_ is the intensity of the initial monochromatic light, I is the transmitted intensity of monochromatic light, ε(λ) is the molar absorption coefficient, c is the concentration of the analyte, and l is the length of light path. To improve the reliability and reduce cost, the discrete and auxiliary optical devices, such as light-emitting diodes (LEDs) and photodetectors, are usually used to construct the autonomous detection systems.

### 2.1. Nitrate and Nitrite

Among various colorimetric assay chemistries, the Griess assay method has been the mainstay for nitrite analysis for over a century [22,23,24,25,26,27,28,29,30,31,32,33,34]. The mechanism of the Griess assay method is that under acidic conditions, nitrite reacts with sulfanilic acid to produce a diazonium salt, which is then coupled to *N*-(1-naphthyl) ethylenediamine (NED), resulting in pink azo compounds that can be detected at 543 nm. For nitrate determination, nitrate should be reduced to a more reactive nitrite by copperized cadmium [6,23,47], zinc [25], vanadium chloride [7,48], etc.

**Figure 1:**
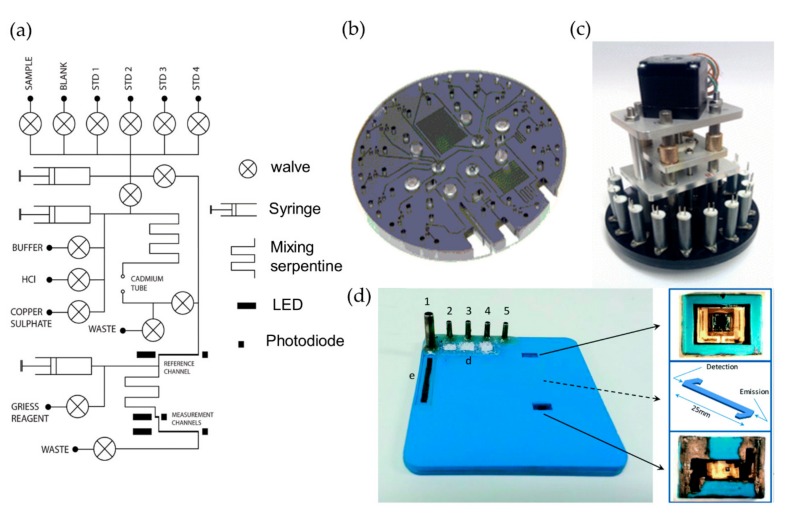
The optofluidic platforms and integrated devices used for the determination of nitrate and nitrite. (**a**–**c**) are the flow diagram, the corresponding photograph, and autonomous integrated device of the microanalyzer used to determine the total nitrate and nitrite. (**d**) The optofluidic chip and the photographs of the autonomous integrated device designed by Cisneros et al. Images reproduced from [6,33].

Figure 1 shows several examples of optofluidic chips and assembled systems using the Griess method for nitrate and nitrite detection. Beaton et al. firstly reported a new generation of miniaturized, in situ, and stand-alone systems with sufficient stability as well as analytical performance for the determination of nitrate and nitrite in natural waters based on optofluidics [33]. As shown in Figure 1a–c, the optofluidic platform consists of a tinted PMMA substrate (5.0 mm thick) micromilled with three absorption cells, three pairs of green LEDs (525 nm), and high sensitivity photodiodes for the detection of nitrate/nitrite with different concentrations. Dark PMMA was used for reducing the background light of LED reaching the photodiode. The optofluidic platform integrated with a custom electronics package for the operational control, data collection, analysis, and transfer. The whole monitoring system has a small size (100 mm diameter and 200 mm height), and low power consumption (about 1.5 W). Autonomous and in situ determination of nitrate and nitrite was deployed with this integrated system, the linear range is up to 350 μM, and the limit of detections (LODs) are as low as 0.025 μM and 0.02 μM for nitrate and nitrite, respectively, making it suitable to be applied in almost all natural waters.

Although the nitrate and nitrite detection systems based on optofluidic technology have been successfully applied in marine online monitoring, there are some drawbacks to be overcome. For example, optofluidic chips are prone to having the problems of bubble formation and blockages due to the narrow microchannels. Martinez-Cisneros et al. developed a compact and automated lab-on-a-chip (LOC) device that integrated microfluidic platform, a highly sensitive colorimetric detection module, and electronics platform for online determination of nitrate and nitrite, as shown in Figure 1d [6]. An embedded and monolithic microcolumn, which was internally installed with copperized cadmium granules, was used as a sample pretreatment module for the reduction of nitrate to nitrite. The device also integrated a bubble removal structure to minimize blockages and avoid potential interferences caused by bubbles, and was successfully applied to the continual monitoring of nitrate and nitrite.

**Figure 2:**
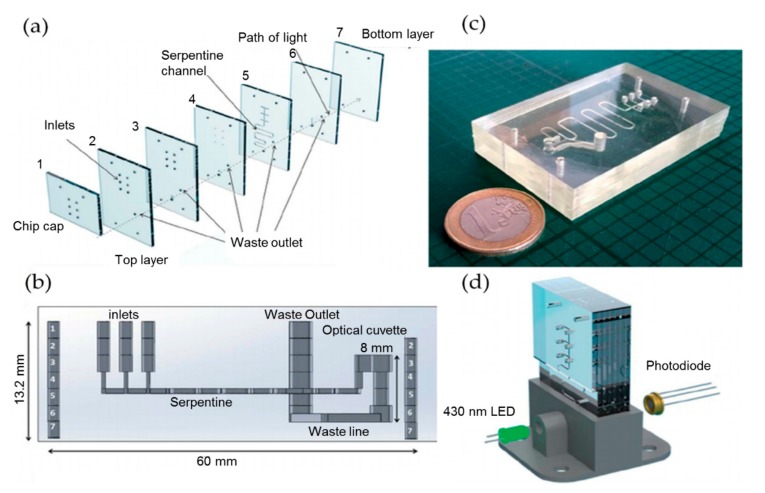
(**a**) Assembly diagram of microchip layers. (**b**) The chip layers that are fully assembled and the micro-cuvette. (**c**) The PDMS microfluidic chip. (**d**) Setup of the microfluidic chip and the optical detection module with LED and photodiode. Images reproduced from [30].

All of the above systems for nitrate detection employ the indirect method, i.e., the concentration of background nitrite is firstly obtained directly after its reaction with the chromogenic agents, the nitrate is then reduced to nitrite, and the second measurement of the total nitrate and nitrite is conducted. The concentration of nitrate finally could be obtained by subtraction. This indirect and time-consuming method adds complexity to the monitoring system. Cogan et al. developed a highly integrated system for nitrate determination. The system was based on the direct and simplified chromotropic acid reagent method, which eliminates the reduction step of nitrate to nitrite [30], as shown in Figure 2. The principle is that the chromotropic acid reacts with nitrate ions in a sulphuric acid medium, resulting in yellow products that can be detected at 430 nm. With this method, a detection range for nitrate from 0.9 to 80 mg/L was achieved with a LOD of 0.73 mg/L. The advantages of simplicity, low cost, low reagent consumption, compact design, and high sample throughput make it an ideal candidate for applying in in situ detection of nitrate.

**Figure 3:**
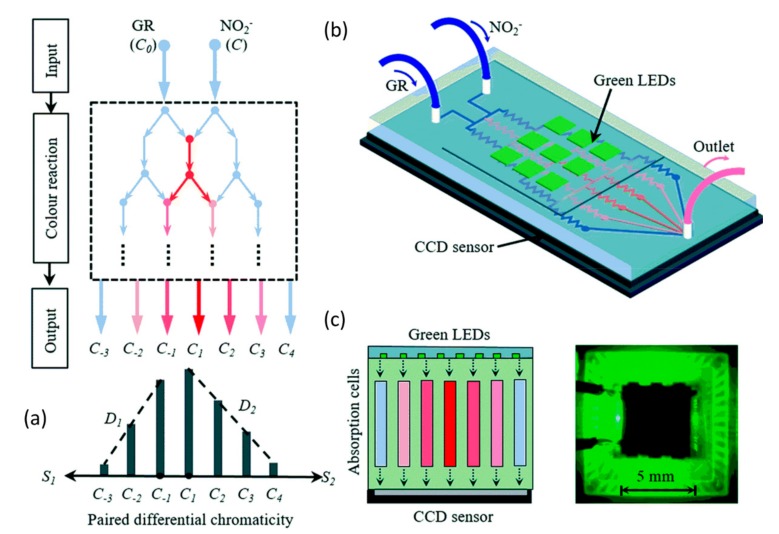
(**a**) Schematic diagram for forming the bidirectional differential concentration. (**b**) Optofluidic platform setup. (**c**) Left: optical detection system with green LEDs and CCD image sensor. Right: physical diagram of the integrated system. Images reproduced from [24].

The traditional colorimetric analysis for nitrate and nitrite detection is time-consuming as it requires determining the calibration curve of the system, which means that a couple of standard samples need to be prepared and detected firstly. To overcome this drawback, some modified approaches based on the Griess assay have been proposed. Recently, Shi et al. demonstrated a robust differential colorimetric method for nitrite detection [24], as shown in Figure 3. The nitrite samples and Griess reagent were pumped into the designed tree-like network on a microchip made from PDMS, forming a unique concentration profile as well as a color gradient network, which contained sufficient information for the determination of nitrite. Only one sample is required for this differential colorimetric method, leading to less consumption of both time and power. The measuring errors for the used two nitrite solutions (0.50 mM and 0.33 mM) are 1.16% and 0.50%, respectively. Compared with the classic method that required a calibration curve, the stability and accuracy of the system are improved by approximately ten times and six times, respectively.

### 2.2. Phosphate

A variety of approaches have been used for the determination of phosphate in seawater, including colorimetric detection, fluorescent detection, and electrochemical detection. All of these are reagent-based methods, as phosphate cannot be detected directly. Generally, autonomous systems tend to employ the colorimetric method rather than fluorescent or electrochemical methods [49,50].

**Figure 4:**
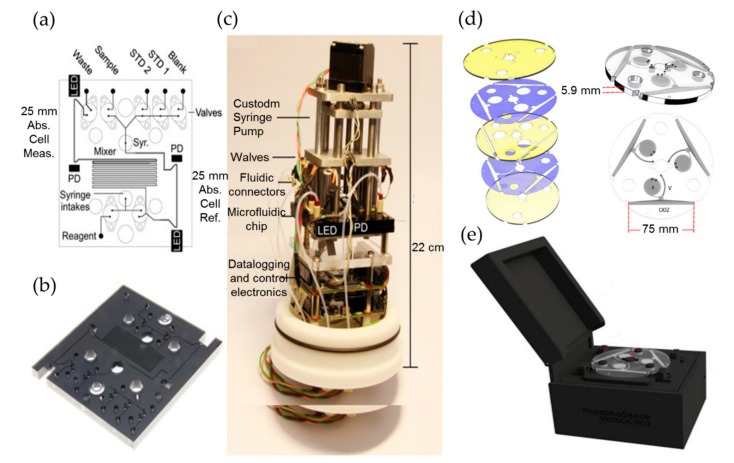
The optofluidic chips and assembled system used for phosphate detection. (**a**–**c**) Flow diagrams, the corresponding photographs, and the autonomous integrated devices of the microanalyzers used to determine the total phosphate ion concentrations. (**d**,**e**) The rendered images showing each layer of the microfluidic disc and the phosphate sense system. Images reproduced from [51,52].

Legiret et al. reported a high-performance optofluidic system for marine phosphate determination using the vanadomolybdate method [51]. The basic principle of this method is that under acidic conditions, orthophosphate reacts with vanadium polymolymolybdate reagent directly and rapidly, resulting in a stable yellow product that can be detected at 375 nm [52,53]. Figure 4a–c shows the optofluidic chip and the assembled system with this approach. The system incorporated an optofluidic chip in tinted PMMA, an optical detection module with high power UV-LEDs as a light source, and photodiodes as absorbance detectors, an embedded control electronics and syringe pumps. The integrated optofluidic analyzer has a small physical size of 22 cm (height) by 10 cm (diameter). Experimental results showed that it has a wide linear range from 0.1 µM to 60 µM and a LOD of 52 nM for phosphate detection. This integrated microanalyzer features high measurement accuracy and resolution, while requires low power and reagent consumption, and it has been successfully deployed in autonomous and in situ phosphate monitoring.

Among all reagent based colorimetric approaches for the determination of phosphate, phosphomolybdenum blue method is the most widely used one [5,51,52,53,54,55,56]. Its principle is that under acidic conditions, orthophosphate reacts with ammonium molybdate, producing a light yellow ammonium phosphomolybdate, which is then reduced by the reducing agent (e.g., ascorbic acid) into a blue compound with strong chromogenic capacity—‘molybdenum blue’. Duffy et al. reported a portable, compact lab-on-a-disc device based on the phosphomolybdenum blue method for in situ quantitation of water phosphate [52]. As shown in Figure 4d,e, the integration of a microfluidic disc allows the use of an optical path length as long as 75 mm for improving sensitivity. The device also integrated a low-cost optical detection system formed by a pair of LEDs and photodiodes. The total mass and the physical size of the device is 2 kg and 20 cm × 18 cm × 14 cm, respectively. The experimental results showed that the detection range for phosphate is 14–800 μg/L, and the LOD is as low as 5 μg/L. Grand et al. reported a newly developed LOC analyzer for long-term and in situ phosphate monitoring [56]. To obtain the best analytical sensitivity, the influence of sample temperature and salinity on the reliability and accuracy was studied, and the reaction parameters were evaluated and optimized. The analyzer is a viable candidate to be deployed in ocean phosphate monitoring as it owns many merits such as perfect stability and robustness, small size, simple to operate, and low energy consumption (1.8 W). It also features a LOD of 30 nM and a wide linear range from 0.05 to 13 µM, with a precision less than 10%.

**Figure 5:**
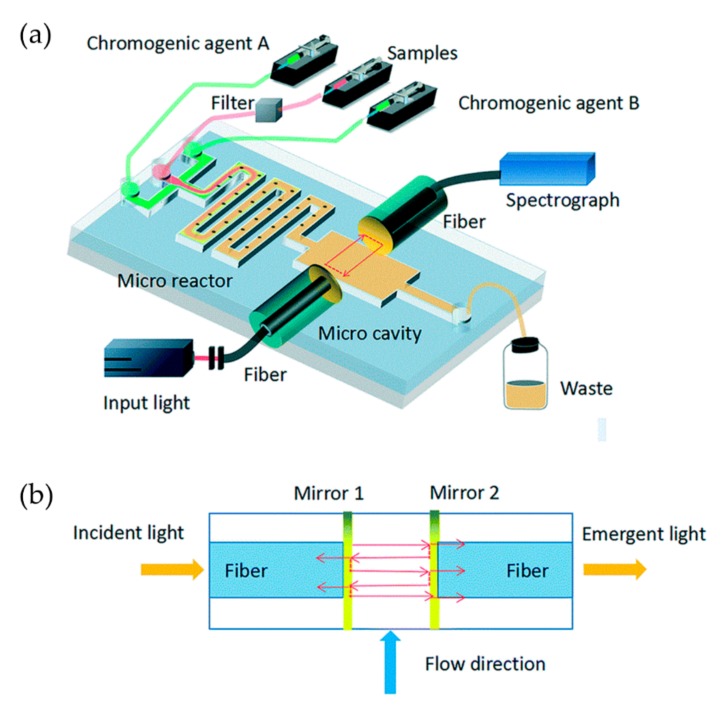
A Fabry–Pérot resonator-based optofluidic device used for monitoring of phosphate in oceans. (**a**) Setup of the optofluidic device. Microchannels with crescent batteries are designed for rapid and enough mixing of regents. (**b**) The microcavity is formed by a pair of optical fibers, on which surfaces are coated with gold. Images reproduced from [5].

For many optofluidic autonomous monitoring systems based on the colorimetric method, the LOD is not comparable to the results obtained by traditional methods because of the limitations on the size of the absorption cell on microchips. To enhance the phosphate absorption and obtain better measurement accuracy, Zhu et al. proposed an innovative on-chip Fabry–Pérot microcavity, which consists of two parallel reflectors made by a pair of fiber facets coated with Au films (Figure 5) [5]. Light is reflected many times in the microcavity, thus lengthening the optical path and enhancing absorption. The length of the absorption cell is shortened from several centimeters to 300 μm, and the device still features a LOD as low as 0.1 μΜ for phosphate detection. Combining the specially designed passive microreactor with semilunar barriers for rapid and enough chromogenic reaction, the required time for detection is shortened from several minutes to six seconds.

### 2.3. Silicate

Silicomolybdenum yellow method and silicomolybdenum blue method are the two standard methods that are commonly used for detection of silicate in seawater. The former method is rapid but features poor sensitivity and is easily interfered with by salinity, making it unsuitable for low concentration analysis. The silicomolybdenum blue method is widely used for the determination of marine silicate as it has the advantage of high sensitivity. Its mechanism is based on the reaction of silicate with ammonium molybdate to form a yellow silicomolybdate complex, which is further reduced to a stable and detectable blue silicomoIybdenum product by ascorbic acid. Cao et al. reported a new generation of optofluidic sensor that has been successfully applied for analyzing the silicate in seawater [57]. The silicate sensor contained four functional modules, including the fluid driving system, a microchip made of tinted PMMA for the mixing and reaction of reagents, a control circuit, and a sensor peripheral. Three absorbance cells were designed for a wide detection range, and three pairs of 810 nm LEDs and photodiodes were used accordingly to perform spectrophotometry analysis. The LOD of the sensor was as low as 45.1 nM, and the linear determination range of the sensor was 0 to 400 μM. An offshore experiment proved that this optofluidic sensor has the advantages of low regent consumption, high accuracy, and robustness.

## 3. pH

Human activities produce a large amount of carbon dioxide (CO_2_). CO_2_ released into the atmosphere could be absorbed by the ocean, leading to significant chemical changes like ocean acidification. The absorption of CO_2_ also regulates the pH of seawater, which is a key parameter for the aquatic organism and influences the ecosystems and biogeochemical cycles of ocean [8,58,59,60,61,62]. A water environment with pH from 6.5 to 8.0 is required for aquatic life, the quantity of water-based life reduces outside of this range because the physiological systems that organisms lived by are affected. Toxic heavy metals (Cd, Pb, Hg, etc.) become more soluble at low pH, thus increasing the toxicity levels of the living environment for organisms [62]. As a result, accurate and timely determination of pH is dispensable for fully understanding the marine carbon cycle and changes of the ecosystem [4,63,64].

Glass electrode method is one of the most popular methods used for pH measurement; accurate results could be achieved if the glass electrodes are maintained and operated properly. However, the glass electrode method also has several drawbacks, such as physical fragility, leakage of the reference electrode buffer, and various responses of the glass electrode with salinity and temperature. All of these disadvantages limit its applications for long-term ocean monitoring. The principle of the most commonly used colorimetric detection of pH is adding indicator dyes, such as thymol blue [65,66], phenol red [67], cresol red [68], and meta-cresol purple [69] to seawater samples, resulting in colored compounds that indicate the pH value.

There are a few examples of optofluidic devices for in situ colorimetric pH measurements. Florea et al. developed a low cost and accurate optofluidic device for pH determination based on polyaniline (PAni), the detectable range for this device is from pH 2 to 12 [70]. The device integrated PAni-based coatings and spectrophotometry method to measure pH. The absorbance spectrum of PAni coatings changes when solutions at various pH values flow along the microchannel. This microfluidic sensor requires no more indicator reagent, thus reducing the complexity of pH detection. Besides, this work evaluated the feasibility of using a digital color camera (e.g., mobile phone with integrated digital cameras) instead of a spectrophotometer to perform colorimetric analysis, which will dramatically extend its applications [71,72]. However, the system is not fully autonomous and requires manual input, like taking photographs and sampling.

**Figure 6:**
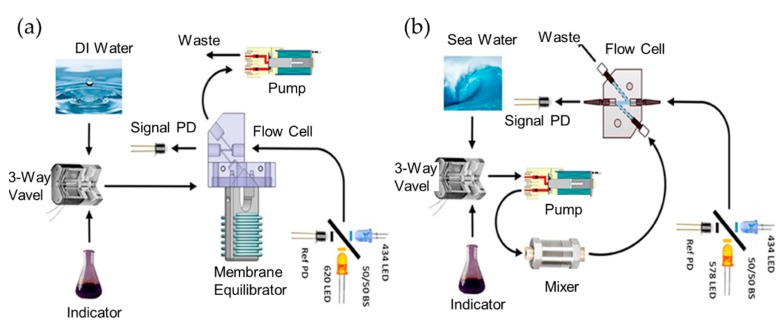
(**a**) Schematics of the detection system for SAMI-CO_2_. (**b**) Schematics of the detection system for SAMI-pH. Images reproduced from [17].

More fully developed autonomous systems for seawater pH measurement have been reported. Rérolle et al. reported a colorimetric pH sensor based on optofluidics for autonomous ocean monitoring. The pH indicator dye of thymol blue was employed in this case [66]. The optofluidic chip is made of tinted PMMA, it consists of a static mixer formed by a serpentine channel as long as 2.2 m, and an absorption cell with a size of 700 μm (length) by 700 μm (width). Its principle is that samples and indicator dye solutions were effectively mixed in the static mixer, then the mixer flows into the absorption cell for optical detection. The detection module consists of a light source with a tricolored LED and a detector with a linear array photodiode spectrometer. The system was successfully implemented in shipboard deployment and a precision of 0.001 pH unit for short-term pH monitoring could be obtained. Lai et al. designed an autonomous optofluidic chemical analyzer for both pH and partial pressure of CO_2_ (pCO_2_) [17]. A pH indicator was used for the determination of the pCO_2_. The indicator was pumped into the gas permeable membrane and then given an optical response when reacting with pCO_2_. With the beam-splitter design shown in Figure 6, the analyzer has a precision of ~±0.5 µatm for pCO2 and ~±0.0005 pH for pH detection. With the specially designed regent bag and adequate power supply, over 8500 measurements could be obtained for every scuba diving. However, the primary defect of this optofluidic analyzer is that it is more complicated compared to other pH and pCO_2_ sensors, such as the single-ended electrode or optode sensor.

## 4. DO

Dissolved oxygen is an indispensable substance for the aquatic organism and a vital parameter to characterize the metabolism of marine ecosystems [73,74,75]. The DO content in seawater is influenced by biochemical and physiological activities. As the DO content of polluted seawater is lower than that of natural seawater, the determination of DO also helps to evaluate the hypoxia and pollution in marine environments.

Many methods have been used to detect DO in oceans, including the iodometric titration method, which is internationally recognized as a benchmark [76], the most widely used electrochemical method, and the optical method, which is mainly based on the principle of fluorescence quenching. Each method has its own merits and drawbacks. The iodometric titration determination has the advantage of high accuracy, but it features cumbersome detection procedures and is not suitable for continuous online detection [77]. Electrochemical method characterizes for rapid detection and simple operation, but it has limitations such as the requirements for calibration and regular maintaining, and the electrode is easily poisoned. The optical DO sensor based on fluorescence quenching has the advantages of versatility and high sensitivity, as well as low toxicity, but the detection accuracy is still needed to improve [78].

Innovative research on the applications of optofluidics in DO detection have been reported. Mahoney et al. proposed a multilayer optofluidic device based on measuring the fluorescence quenching in a Ruthenium-based oxygen sensitive dye. Enhanced sensitivity was achieved by employing total internal reflection (TIR) of the excitation light. The optofluidic sensor exhibits high sensitivity for the detection of 0–20 ppm DO in water [79]. However, the automation and miniaturization of DO sensors for ocean monitoring based on optofluidic technology remain to be developed [80,81].

## 5. Heavy Metals

It is of great importance to monitor heavy metals in oceans as they can seriously affect the environment and human health. Generally, heavy metal refers to metals with specific gravities greater than 5 g/cm^3^. It includes essential metals that are indispensable for the normal physiological activities of organic life such as iron (Fe); copper (Cu); magnesium (Mg); selenium (Se); zinc (Zn); manganese (Mn); nonessential metals including cadmium (Cd), mercury (Hg), silver (Ag), lead (Pb), and gold (Au); and some unusual metals with high atomic weight [82]. With the accumulation of essential metals in organisms, toxic side effects will occur if its concentration exceeds a certain threshold [83]. Nonessential metals could be hypertoxic even at a trace level, although they do not participate in the metabolic activities of organisms.

Many analytical techniques have been proposed for the determination of heavy metals in seawater, for example, the inductively coupled plasma mass spectroscopy (ICP-MS) method and atomic absorption spectrometry method. However, both of them require tedious sample preparation and pretreatment, expensive equipment, and professional personnel, making them insuitable in applications for autonomous, in situ, and continuous monitoring of heavy metals. In this case, ocean monitoring sensors based on colorimetric, fluorescent, and chemiluminescent methods appear as promising technologies due to the high sensitivity and feasibility, versatility, and reproducibility [84,85,86,87,88,89,90,91,92,93,94,95], and all of these methods have been truly applied for online analysis of heavy metals in seawater.

**Figure 7:**
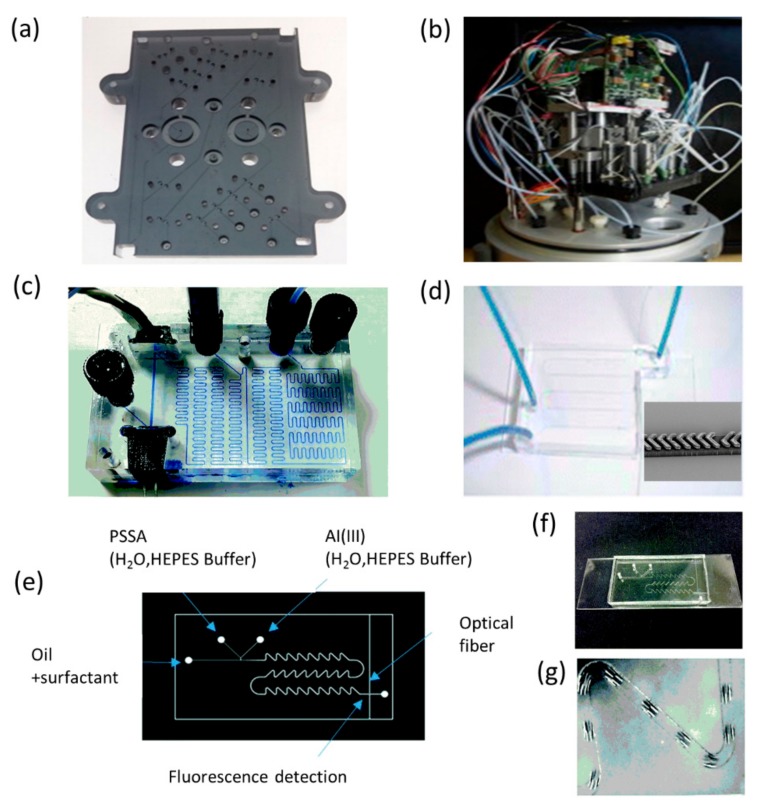
Spectrophotometric and fluorescent detection of heavy metal by optofluidic systems. (**a**,**b**) The photograph and the autonomous integrated device of the microanalyzers used to determine dissolved Fe and Mn with a spectrophotometric method. (**c**) The PMMA optofluidic chip used for arsenic detection with a colorimetric method. (**d**) The photograph of the optofluidic chip for fluorimetric detection of cadmium. (**e**) The scheme of the optofluidic set-up following the droplets approach for fluorescent detection of water-soluble aluminium. (**f**,**g**) The photographs of the microchip and the forming droplet of the fluorescent sensor. Images reproduced from [88,91,92,102].

The colorimetric method for heavy metal monitoring is also based on Lambert–Beer’s law. The principle is that under certain conditions, heavy metal ions react with a specific reagent, producing a new colored chemical solution, and the absorbance of the solution is related to the concentration of the heavy metal. Different heavy metals need different color developing agents when employing the colorimetric method. For example, silver salt is used for arsenic determination [96], dithizone is generally used for lead and zinc determination [97], while dimethylglyoxime is generally applied for nickel determination [98]. Milani et al. developed an autonomous LOC colorimetric system with high performance and low-cost for the in situ monitoring of dissolved Fe(II) and Mn in natural water [88]. The optofluidic platform consists of a microfluidic chip made of PMMA and two pairs of LEDs and photodiode detectors for detection of Fe(II) and Mn, as shown in Figure 7a,b. With this portable device, 12 samples of Fe(II) and 6 samples of Mn could be analyzed per hour with LODs of 27 nM and 28 nM, and precisions of 2.1% and 2.4% (*n* = 19), respectively. Lace et al. reported an innovative optofluidics system for monitoring of arsenic in water with the leucomalachite green dye method [91]. The principle involves the reactions of arsenic and potassium iodate under acidic conditions, and leucomalachite green is oxidized to malachite green by the liberated iodine, producing a green color with an absorption peak at 617 nm. Chromogenic agents and water samples are mixed and reacted in a microfluidic chip, as shown in Figure 7c. The leucomalachite green method was optimiszd and water arsenic within the range of 0.3–2 mg /L could be detected, the LOD of the system was 0.32 mg/L, and the average relative standard deviation (RSD) was 21.1%.

The fluorescent method is an excellent option for autonomous and in situ ocean heavy metal detection. Compared with the colorimetric method, higher sensitivity and lower LOD could be achieved with the fluorescent method. The basic mechanism of the fluorescent method is that the fluorescence parameters (such as fluorescence intensity, lifetime, and spectrum) change in response to the concentration variations of some ions. Until now, numerous fluorescent sensors based on chelation-enhanced fluorescence [98,99] photo-induced electron transfer [92], aggregation-induced emission effect [100], and intramolecular charge transfer [101] have already been reported for heavy metal monitoring. Leray’s group developed several optofluidic devices that incorporated fluorimetric detection for the monitoring of heavy metals such as aluminum and cadmium [92,93,102]. Recently, the aluminum-sensing mechanism by PSSA–4-propoxysulfonate salicylaldehyde azine was proposed [92]. As shown in Figure 7e–g, the sensing system includes a microfluidic chip made from PDMS/glass, and a fluorescence detection platform consists of a LED (365) nm and a photomultiplier (PMT) detector. In brief, the PSSA and Al(III) are injected and mixed in a Y-shaped junction, the complexation of the ligand with the analyte occurs upon mixing, producing a fluorescence signal, which is collected and detected by the PMT. Regularly monodispersed and spaced microdroplets are formed, and a highly reproducible and reliable monitoring environment was provided as the system to adopt the microfluidic droplet technology. The experimental results showed that the system has a LOD of 153 nM. The newly designed fluorescent sensor for cadmium detection adopts a commonly used water-soluble commercial dye Rhod-5N, whose fluorescence intensity increased with the existence of toxic cadmium. The optofluidic device is made from PDMS chip and a glass substrate, as shown in Figure 7d. The famous staggered herringbone structure [98,102] was used to form a passive mixer for the thorough mixing of the Rhod-5N reactant and the Cd^2+^ analyte. The optofluidic device obtained a promising LOD of 0.45 μg/L in 3-(*N*-morpholino) propanesulfonic acid buffer at pH 7.

The chemiluminescent method for quantitative analysis of heavy metals is mainly based on the linear relationship between the concentration of the analyte and the chemiluminescence intensity of the system under certain conditions. The most commonly used chemiluminescent reagents include luminol, acridine esters, and 1,2-dioxygen cycloethane. Based on the chemiluminescent reaction of Mn contained in the seawater sample with a luminol-based reagent, Christophe et al. developed an integrated in situ analyzer for detecting Mn (IISA-Mn) in the environment of the deep sea [89]. A miniaturized PDMS device with Tesla mixer structures was used to enhance the mixing and reaction efficiency, and a PMT detector was used to measure the chemiluminescent intensity. As a result of the integration of fluidic elements such as mixers, valves, and flow regulators, this analyzer based on optofluidics occupies less volume (3 L) and consumes less reagent (<130 mL/24 h) compared with its macroworld counterparts. The IISA-Mn gives a linear range of 0 to 500 nM and a LOD of 280 nM in seawater, making it sufficient for detecting geochemical anomalies such as hydrothermal ore deposition surveys. The system has been proven to be able to work flawlessly and continuously during the 8 h of an actual remotely operated vehicle dive.

## 6. Discuss and Outlook

There is more and more research on the optofluidic devices as optofluidic technology has advantages of low cost, small size, low reagent, and power demands. Although we emphasize the applications of optofluidic platforms for the monitoring of chemical parameters in ocean environment in this review, they could also find uses in biological monitoring in the marine ecosystem. For example, a variety of in situ bio/biochemical analyzers (e.g., adenosine triphosphate analyzer and gene analyzer) have been developed on optofluidic platforms, most of them have been evaluated in the real ocean environments including the deep sea [103,104].

As marine environmental pollution is increasingly severe, there is a growing demand for the further development of autonomous and in situ ocean monitoring. To realize continuously offshore monitoring with high practicability, stability, and credibility, smaller and lighter portable instruments with less power consumption and higher accuracy are needed. Optofluidic technology is one of the most promising approaches to realize the miniaturization and automation of ocean monitoring. This paper reviewed the optofluidic devices integrated with microelectromechanical systems that are applied for in situ ocean monitoring; up-to-date developments of optofluidics for autonomous ocean environmental monitoring were covered. Optofluidics has the challenge of ensuring that the overall system can achieve the required sensitivity for ocean applications where analytes exist at only trace levels. However, we firmly believe that based on the development trend of full automation and miniaturization of marine monitoring equipment, and with the development of the supported technology such as three-dimensional printing, inkjet printing, power-free pumping and micromachining technologies, the low-cost lab-on-chip systems based on optofluidic technology will inspire a promising future in the field of ocean observations.
